# Village and farm-level risk factors for avian influenza infection on backyard chicken farms in Bangladesh

**DOI:** 10.1038/s41598-022-16489-5

**Published:** 2022-07-29

**Authors:** Suman Das Gupta, Brishti Barua, Guillaume Fournié, Md. Ahasanul Hoque, Joerg Henning

**Affiliations:** 1 School of Veterinary Science, University of Queensland, Gatton, QLD 4343 Australia; 2 Chattogram Veterinary and Animal Sciences University, Chattogram, Bangladesh; 3 Department of Pathobiology and Population Sciences, Royal Veterinary College, University of London, London, UK

**Keywords:** Risk factors, Diseases, Infectious diseases, Policy and public health in microbiology, Disease prevention, Public health

## Abstract

A cross-sectional study was conducted with 144 small-scale poultry farmers across 42 Bangladeshi villages to explore risk factors associated with avian influenza H5 and H9 seropositivity on backyard chicken farms. Using mixed-effects logistic regression with village as random effect, we identified crow abundance in garbage dumping places and presence of migratory wild birds within villages to be associated with higher odds of H5 and H9 seropositivity. At farm-level, garbage around poultry houses was also associated with higher odds of H5 and H9 seropositivity. In addition, specific trading practices (such as, purchase of chickens from live bird markets (LBM) and neighboring farms to raise them on their own farms, frequency of visits to LBM, purchase of poultry at LBM for consumption) and contact of backyard chickens with other animals (such as, feeding of different poultry species together, using pond water as drinking source for poultry, access of feral and wild animals to poultry houses) were associated with higher odds of H5 or H9 seropositivity. Resource-constrained small-scale poultry farmers should be able to address risk factors identified in this study without requiring large investments into poultry management, thereby reducing the likelihood of avian influenza virus transmission and ultimately occurrence of avian influenza outbreaks.

## Introduction

Backyard chickens reared under traditional scavenging conditions are an essential source of nutritious food^1^ and income for rural households in low and middle-income countries (LMICs) like Bangladesh^2,3^. However, the low level of biosecurity in this farming system may put backyard poultry at a high risk of infection with avian influenza viruses (AIVs)^4^. Backyard poultry is therefore often considered to promote the spread and persistence of AIVs^5,6^. On the other hand, it has been hypothesized that, due to their small flock size, the risk of a viral introduction and spread may be substantially lower for backyard compared to commercial flocks^7,8^, and that local breeds, raised on backyard farms, are less susceptible to infection than exotic breeds reared in commercial enterprises^9,10^. However, there is no experimental and observational evidence supporting these hypotheses^11^.

Highly Pathogenic Avian Influenza (HPAI) H5N1 was first reported in Bangladesh in 2007. It is now endemic in Bangladesh with multiple AIV subtypes, including Low Pathogenic Avian Influenza (LPAI) H9N2, circulating in the country’s poultry population, raising concerns about the emergence of a new AIV variant with significant public health concern^12,13^. A recent study conducted in Bangladeshi Live Bird Markets (LBM) estimated a prevalence of H5 and H9 AIVs of 1.3% and 8.3% in backyard chickens, 7.6% and 3.4% in waterfowl (ducks and geese), respectively^14^. In contrast, in another field study conducted by Gupta et al.^15^, none of the chickens and ducks sampled on backyard flocks tested positive for H5 AIV, and only 0.2% of chickens tested positive for H9 AIV^15^. The H5 and H9 seroprevalence in this study was 4.2% and 16.0% in unvaccinated backyard chickens, and 14.2% and 15.7% in unvaccinated ducks, respectively, indicating past exposure of backyard chicken to circulating H5 and H9 virus^15^.

The implementation of comprehensive biosecurity practices is notoriously challenging, if at all feasible, in backyard chicken farming systems^16^. The lack of adherence to recommended biosecurity practices is also likely influenced by backyard farmers’ beliefs that their poultry does not play a significant role in AIV transmission^5^. It is therefore essential to identify risk factors contributing to AIV infection, in order to develop specific extension messages and recommendations for improved biosecurity that are low-cost and feasible for backyard poultry farmers. To our knowledge, only one case–control study was conducted in Bangladesh more than 13 years ago, that focused on the identification of farm-level factors associated with the occurrence of H5N1 outbreaks on backyard farms^17^. Risk factors for current H5 and H9 circulation in apparently healthy backyard poultry have not been described in Bangladesh.

In this study, we focused on clinically healthy chickens to monitor their past exposure to AIV. By focusing on chickens without clinical signs, we can describe the circulation of AIV in chickens that might had occurred in these flocks and risk factors associated with this AIV exposure. We are also able to inform farmers about potential threats to their current poultry production so that they can take appropriate actions. Furthermore, as the co-infection of the HPAI virus with other viruses poses a danger for the occurrence of more severe avian influenza disease in poultry and in humans, identifying risk factors for AIV exposure in clinically health birds provides an opportunity for implementing early interventions. Focusing the data collection on clinically healthy chickens does also allow for random sampling, as targeting clinically-affected birds would have likely lead to selection bias.

Therefore, this research study aimed to identify farm and village-level factors relating to the management and marketing of backyard chickens, as well as environmental or ecological conditions, village structure, poultry density, past disease outbreaks and past vaccination campaigns that might be associated with H5 and H9 seropositivity in backyard chickens. We hypothesize that risk factors do exist that can be easily addressed and modified in order to reduce the likelihood of AIV transmission and ultimately avian influenza outbreak occurrence.

## Results

None of the sampled backyard chicken flocks were vaccinated against AI. Farmers did not report any outbreaks of HPAI or unusually high mortality in their flocks within the last 12 months preceding the sampling.

We used the farm-level H5 and H9 serological status as a binary outcome variable (H5/H9 seropositive or seronegative): a farm/flock was considered positive for a given AIV subtype if at least one chicken or duck on that farm/flock had a Haemagglutination Inhibition (HI) titre of ≥ 2^4^^18^.

The proportions of H5 and H9 seropositive flocks were 27.8% (N = 40) and 60.4% (N = 87), respectively. The residual Intra-class Correlations (ICCs) were 0.11 (95% CI 0.00–0.87) and 0.18 (95% CI 0.02–0.75) for H5 and H9 seropositivity, respectively, suggesting significant clustering at the village-level.

In the univariate analysis, 7 village-level and 10 farm-level risk factors associated with H5 seropositivity were identified (Table 1, Supplementary Table S1), while 7 village-level and 14 farm-level risk factors were associated with H9 seropositivity (Table 2, Supplementary Table S2). The final multi-variable model for H5 AIV exposure included one village-level and four farm-level risk factors (Table 1), while for H9 AIV exposure, it comprised of two village-level and five farm-level risk factors (Table 2).

**Table 1:** Results of the univariate and multi-variable analysis for village and farm-level risk factors (N = 144 farms, N = 42 villages) associated with H5 flock-level seroprevalence on backyard chicken farms in Bangladesh, 2016.

Risk factors (listed in risk groups)	Category	Univariate analysis				Multi-variable analysis	
		H5 positive (%)	H5 negative (%)	H5 OR (95% CI)	H5 *p *value	H5 OR (95% CI)	H5 *p *value
**Village-level factors (N = 144 farms, N = 42 villages)**							
*Environmental or ecological features*							
Crow abundance around a garbage dumping places within the village	No crows or absence of garbage dumping place	20 (19.6)	82 (80.4)	Reference	**0.001**	Reference	**0.039**
	Yes	20 (47.6)	22 (52.4)	3.7 (1.7–8.1)		3.4 (1.1–10.8)	
**Farm-level factors (N = 144 farms)**							
*Trading practices*							
Number of chickens bought from live bird markets in the last 12 months	0	25 (20.5)	97 (79.5)	Reference	**0.000**	Reference	**0.016**
	1–3	6 (54.6)	5 (45.5)	4.7 (1.3–16.5)		9.5 (1.3–69.9)	
	> 3	9 (81.8)	2 (18.2)	17.5 (3.5–86.0)		8.8 (1.2–65.9)	
*Disposal of garbage*							
Garbage piled up around the poultry houses or on the farm	No	23 (19.0)	98 (81.0)	Reference	**0.000**	Reference	**0.010**
	Yes	17 (73.9)	6 (26.1)	30.8 (5.6–168.7)		9.1 (1.7–48.8)	
*Indirect contact with other animals*							
Feeding of different poultry species with the same feeder or in the same location	No	10 (13.0)	67 (87.0)	Reference	**0.000**	Reference	**0.003**
	Yes	30 (44.8)	37 (55.2)	5.8 (2.4–14.3)		5.2 (1.7–15.7)	
Pond water used as source of drinking water for poultry	No	13 (16.3)	67 (83.8)	Reference	**0.002**	Reference	**0.010**
	Yes	27 (42.2)	37 (57.8)	4.1 (1.7–10.2)		4.6 (1.4–14.9)	

Significant values are in bold.

**Table 2:** Results of the univariate and multi-variable analysis for village and farm-level risk factors (N = 144 farms, N = 42 villages) associated with H9 flock-level seroprevalence on backyard chicken farms in Bangladesh, 2016.

Risk factors (listed in risk groups)	Category	Univariate analysis				Multi-variable analysis	
		H9 positive (%)	H9 negative (%)	H9 OR (95% CI)	H9 *p *value	H9 OR (95% CI)	H9 *p *value
**Village-level factors (N = 144 farms, N = 42 villages)**							
*Environmental or ecological features*							
Crow abundance around a garbage dumping place in the village	No or absence of garbage dumping place	53 (52.0)	49 (48.0)	Reference	**0.004**	Reference	**0.004**
	Yes	34 (81.0)	8 (19.1)	4.3 (1.6–11.5)		13.1 (2.3–76.8)	
Migratory wild birds visiting the village	No	37 (48.7)	39 (51.3)	Reference	**0.006**	Reference	**0.007**
	Yes	50 (73.5)	180 (26.5)	3.1 (1.4–7.1)		5.8 (1.6–21.1)	
**Farm-level factors (N = 144 farms)**							
*Trading practices*							
Live poultry obtained from neighbours in the last 12 months and incorporated into backyard flocks	No	69 (56.1)	54 (43.9)	Reference	**0.025**	Reference	**0.020**
	Yes	18 (85.7)	3 (14.3)	4.7 (1.2–17.9)		8.1 (1.4–46.9)	
Number of live bird market visits by farmers (or households members) in the last month for any purpose rather than selling poultry and eggs	0 times	12 (46.2)	14 (53.9)	Reference	**0.045**	Reference	**0.038**
	1–5 times	64 (61.0)	41 (39.1)	2.3 (0.8–6.4)		3.8 (0.9–16.1)	
	> 5times	11 (84.6)	2 (15.4)	12.7 (1.6–97.2)		47.2 (2.4–933.3)	
Purchase of poultry for consumption from live bird markets and processing on backyard farm	No purchase of poultry for consumption from LBM; or if purchase processing at LBM	8 (38.1)	13 (61.9)	Reference	**0.015**	Reference	**0.021**
	Purchase of poultry for consumption from LBM and processing on backyard farm	79 (64.2)	44 (35.8)	5.1 (1.4–18.7)		9.3 (1.4–62.1)	
*Disposal of garbage*							
Garbage piled up around the poultry houses or on the farm	No	66 (54.6)	55 (45.5)	Reference	**0.003**	Reference	**0.002**
	Yes	21 (91.3)	2 (8.7)	12.3 (2.3–65.8)		28.6 (3.4–239.8)	
*Indirect contact with other animals*							
Holes in the poultry house allowing feral/wild animals to enter	No	23 (45.1)	28 (54.9)	Reference	**0.010**	Reference	**0.001**
	Yes	64 (68.8)	29 (31.2)	2.7 (1.3–5.9)		10.8 (2.8–41.9)	

Significant values are in bold.

At village-level, crow abundance around the garbage dumping place was associated with higher odds of both H5 and H9 seropositivity. Also, migratory wild birds visiting the village increased the odds of H9 infection. At farm-level, garbage piled up around poultry houses (or on the farms in general), was associated with higher odds of both, H5 and H9 seropositivity. Furthermore, the odds of a backyard farm being seropositive for H5 increased with the number of chickens bought from LBMs and raised on these backyard farms, with the feeding of different poultry species from the same feeder or in the same space and with the use of pond water as a drinking source for poultry. In addition, purchasing poultry from neighbouring farms to raise them on backyard farms, the frequency of visits to LBM, the purchase of poultry at LBM for consumption and the access of feral and wild animals to poultry houses increased the odds of a farm being seropositive for H9. We did not observe any confounding effect of initially eliminated variables that were added into the final multi-variable models. We also did not identify any significant 2-way interactions.

## Discussion

The observed H5 seropositivity without any HPAI clinical signs or mortalities might be the result of infection of backyard chickens with LPAI H5 strains. A study conducted by Gerloff et al.^19^ reported the presence of LPAI H5N2 in Bangladesh, and other studies by Duan et al.^20^ and Nguyen et al.^21^ also indicated the circulation of LPAI H5 viruses (H5N2, H5N3, H5N8), in Asia. Alternatively, viral evolution might have resulted in a reduction of HPAI H5 pathogenicity^22^, or the chickens might have developed reduced susceptibility to clinical disease due to cell-mediated immunity^23^.

### Environmental and ecological village-level risk factors

Our study identified crow abundance in garbage dumping places within a village as a risk factor for both, H5 and H9 seropositivity. In Bangladesh, crows have often been found feeding on household scraps and garbage from LBM^24^. The presence of crows in villages may be indicative of locations, where poultry farming-related waste including dead birds and poultry droppings have been disposed. Backyard chickens are likely to scavenge around such locations and might be exposed to AIV-contaminated material. Indeed, rather than being vectors of infection, crows may well act as sentinels to an AIV-contaminated environment and might become infected themselves. Infection of crows by H5N1^25,26^ and H9N2^27,28^ viruses resulting from their exposure to garbage dump has been previously reported.

The presence of migratory wild birds in villages was also associated with an increased odds of farms being positive for H9. The world’s largest delta, the Jamuna-Padma (or Brahmaputra-Ganges) covers most of Bangladesh. These waterways attract many migratory wild birds travelling along the ‘Southeastern end of the Central Asian Flyway’ and the ‘Southwestern end of the East Asian—Australasian flyway’, to overwinter in Bangladesh. Thus, a range of migratory wild bird species are visiting Bangladesh during the winter months^29,30^. Migratory wild birds mingle frequently with domestic waterfowls^31^, which may create opportunities for viruses to be introduced into village poultry populations. Contact between wild birds and backyard poultry was identified as a risk factor for H9N2 seropositivity in backyard poultry farms in Pakistan^32^. Similarly, in Egypt, a study^33^ on the circulation of HPAI and LPAI viruses in backyard chickens highlighted potential interactions of backyard chickens with waterfowl and shorebirds and recommended improved biosecurity practices to mitigate the AIV transmission between wild and domestic birds. In fact, active surveillance data collected from wild birds and backyard flocks in Northern Italy in 2004–2006 confirmed that contacts between migratory birds and free-range backyard poultry are a likely pathway for AIV transmission^34^.

### Farm-level risk factors relating to garbage disposal

Similarly, to the village-level risk factor related to garbage disposal, garbage piled up on farms was also a risk factor for both, H5 and H9 seropositivity. A survey conducted in the United States identified garbage as a possible source of HPAI virus infection for commercial poultry farms^35^. In this study, potentially HPAI-contaminated material (poultry carcasses, eggshells, dead wildlife) was disposed near backyard poultry farms. The study also suggested that a garbage collection service shared by commercial and backyard poultry farmers might have been one of the potential pathways for HPAI virus spread^35^. Considering that AIVs shed into the environment may remain infectious for weeks or months at ambient temperatures, untreated garbage in open areas can play a significant role in the AIV epidemiology^36,37^. However, extreme seasonal variations in rainfall, humidity and temperatures (as observed in Bangladesh) might impact the survival of AIV in farming environement^38^.

### Farm-level risk factors relating to trading practices

Purchase of poultry at LBMs for raising them in farmers’ own backyard flocks or for consumption was associated with increased H5 and H9 seropositivity. AIVs have been found to circulate with high prevalence in LBMs of countries, including Bangladesh, where live bird trading is a common practice^39^. Purchasing poultry from neighbouring farms was also found to increase the odds of a farm to be positive. A recent cross-sectional study conducted by Chaudhry et al.^40^ exploring farm-level risk factors associated with AI seropositivity across 308 villages of the Lahore district in Pakistan also found that backyard poultry farms, that purchased birds from LBM or obtained them from friends had increased odds of AI seropositivity. Similar observations were made in Thailand, further emphasizing the importance of the poultry trade in the spread of AIVs^41^.

### Farm-level risk factors relating to contact of backyard chickens with other animals

Husbandry practices such as using the same equipment to feed multiple species of poultry together, promoted inter-species contacts and thereby increased the odds of a farm being seropositive for H5. In addition, the use of pond water as a drinking source also increased the odds of both H5 and H9 seropositivity. In Bangladesh, ponds represent shared habitats for domestic and migratory waterfowls, and water may be contaminated with AIVs. Indeed, an experimental laboratory-based study reported that H5N1 virus can remain infectious in water for 12 days at 22–35 °C^42^. Ponds might also become contaminated with AIV through the disposal of dead poultry, which is a common feed source for farmed fish in Bangladesh. Moreover, access of feral or wild animals to poultry houses, identified here as a risk factor, has been previously described as a plausible route for H9 virus transmission^43^. A review study pointed out the role of wild and terrestrial animals in the potential replication and shedding of AIVs, highlighting that these animals are able to replicate and shed high-titres of multiple AIV subtypes without any prior viral adaptation^44^.

### Study limitations

There are some limitations to our study. Firstly, as in any research involving interviews, recall bias may have affected farmers’ responses. However, as we specifically interviewed the people who actually care for the chickens, we are confident to have minimised this bias. Secondly, due to the cross-sectional nature of the study, we could not assess the impact of seasonal variations on the predictors. Thirdly, interviewees may have given socially acceptable answers to some sensitive questions, for example on questions related to cleaning and disinfection practices. This may explain why those variables were not identified as significant risk factors in the final multi-variable model. Fourthly, chickens infected by HPAI H5 are expected to die, although backyard farmers did not report any unusual mortalities or HPAI outbreaks on their farms over the year preceding the sampling. Being unable to confirm this, we trusted the farmers observations. Finally, we used seropositivity as a measure of past exposure of backyard chickens to AIV. Although, we also collected swabs from birds to assess virus shedding, virus prevalence was very low (flock-level H9 virus prevalence was 0.7% and no flocks were positive for H5) and we were not able to use it as an outcome variable in the risk factor analysis.

## Conclusions and recommendations

The focus of this study was on identifying practical solutions that can be addressed by resource limited small-scale backyard chicken farmers without any large additional investments into poultry management. Thus, the results of this research lead to the following conclusions and recommendations that can reduce the risks of AIV infection in backyard poultry flocks:

- Garbage should be disposed as far as possible from farms by burning or burying it deep in the ground, so that scavengers, including backyard poultry, cannot access it. Backyard farmers need to be encouraged to avoid dumping of poultry droppings or even dead birds in waste areas within villages and on their farms as this might attract wild birds (e.g. crows).
- Backyard farmers who rear multiple poultry species within the farm should be discouraged from feeding different species of poultry using the same feeder or trough.
- Alternative water sources, such as tube-well water, should be used to provide water to backyard poultry.
- Backyard farmers should be encouraged to purchase poultry from reliable sources to supplement their own flocks. In particular, sources of live poultry with likely contact with infected birds (e.g. poultry at LBM) should be avoided, or at least newly purchased birds should be separated from the rest of the flock for a minimum of two weeks. Backyard farmers should be encouraged to hatch their own birds in a low-cost and feasible bio-secured environment.
- Live poultry bought from LBM for family consumption should not be slaughtered and/or processed at home, but at the LBM.
- As much as practical and feasible, the movement of backyard farmers or their family members to LBM should be minimized.
- Backyard farmers should be encouraged to restrict the scavenging of their poultry in waste areas.

We believe that the risk factors identified in this study can also help policy makers to develop more specific and practical biosecurity measures aiming to mitigate the risk of AIV infection in backyard chickens.

## Methods

### Overview of the study design

A cross-sectional study was conducted in Chattogram (previously, Chittagong) and Cox’s Bazaar districts of Bangladesh in February and April 2016. Chattogram is the second largest city in Bangladesh after the capital Dhaka. About 19.7% of the country’s urban population lives in Chattogram, and the city contributes 30% of the country’s national Gross Domestic Product^45,46^. Most chickens sold through Chattogram City Live Bird Markets are supplied by Chattogram and Cox's Bazar districts^47,48^. About 0.7 and 0.3 million households in the Chattogram and Cox’s Bazar districts, respectively^49,50^, raise backyard poultry, which are an important subsistence income for this rural population^51^. Considering the significance of poultry production and trade in these two districts, we used a list of backyard chicken farms within these two districts as our target population. The study was conducted on 144 backyard chicken farms across 42 villages in these two districts. The sample size estimation and the selection of study units have been described in detail in Gupta et al.^15^.

### Questionnaire data

**Figure 1:**
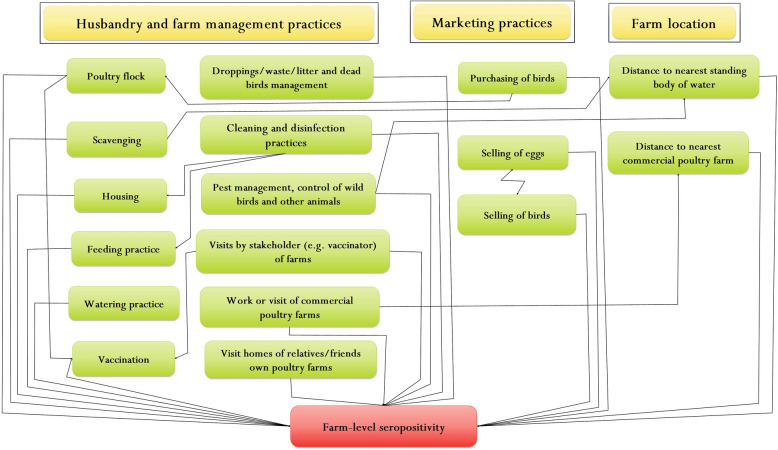
Hypothesized causal pathways for farm-level risk factors (green boxes) associated with avian influenza farm-level seropositivity (red box) in backyard chickens in Bangladesh. Yellow headings represent themes or categories under which risk factors can be combined. The causal pathways were used to inform the development of questions used in the interviews with backyard farmers and guided the inclusion of potential confounders and interactions in the final multi-variable model.

**Figure 2:**
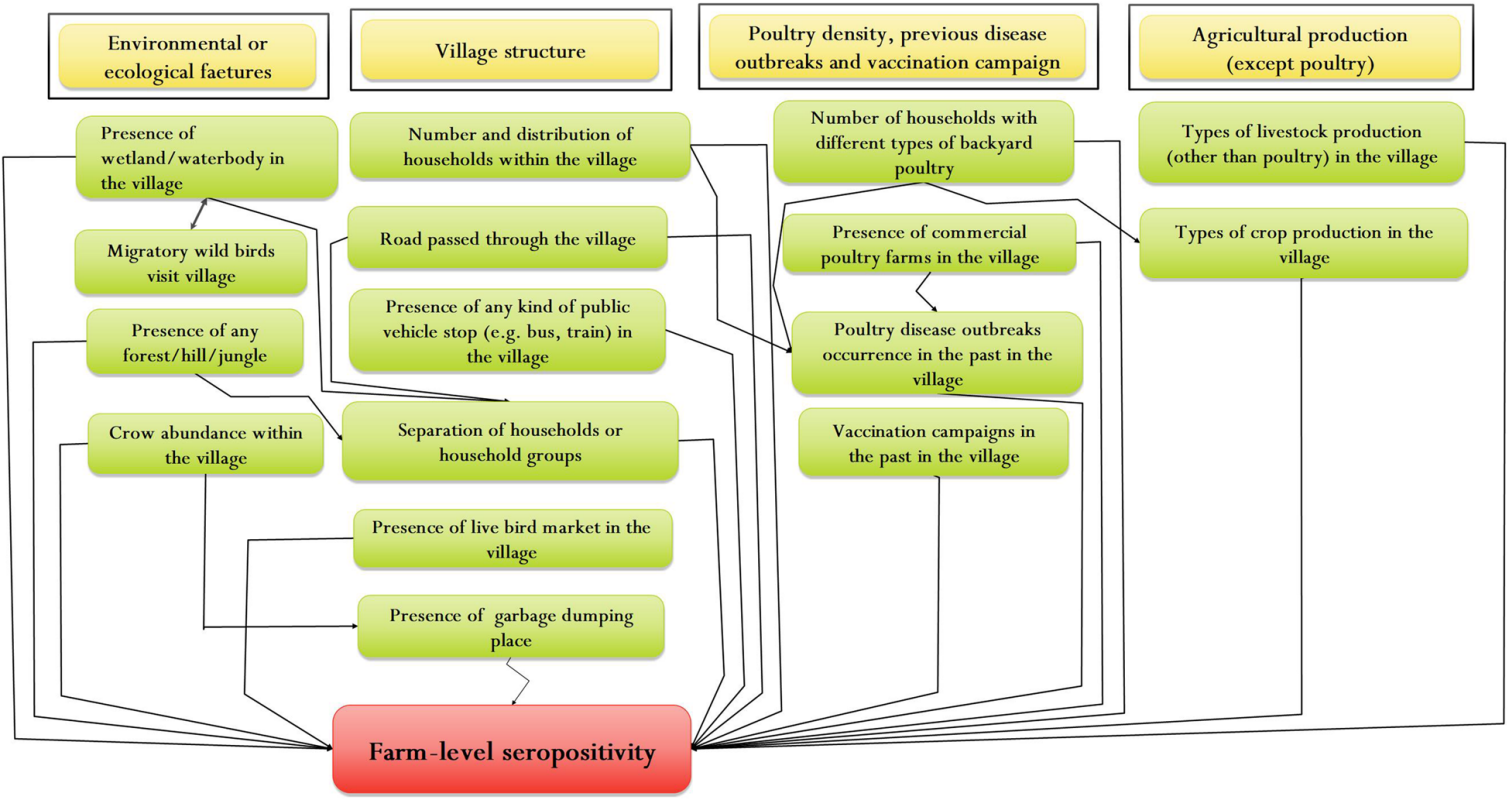
Hypothesized causal pathways for village-level risk factors (green boxes) associated with avian influenza farm-level seropositivity (red box) in backyard chickens in Bangladesh. Yellow headings represent themes or categories under which risk factors can be combined. The causal pathways were used to inform the development of questions used in the interviews with backyard farmers and guided the inclusion of potential confounders and interactions in the final multi-variable model.

We used two types of questionnaires to collect information on farm-level and village-level risk factors potentially associated with AIV circulation. Both questionnaires were developed in English and then translated into Bengali language. The questionnaires were developed based on causal diagrams constructed using the software MindMaple Lite version 1.3 (MindMaple Inc., Tustin, USA) visualising the hypothesized relationships between the flock-level serological status and potential farm (Fig. 1) and village-level risk factors (Fig. 2). The detailed causal diagrams for risk factors at farm and village-level are shown in Supplementary Figs. S1 and S2, respectively.

The farm-level questionnaire was designed to capture information on husbandry and marketing practices, and on the farm location. The questionnaire was pilot-tested on five backyard farms that were not part of the farms recruited for the study. Twelve out of 58 questions were subsequently modified as their initial formulation was unclear for interviewed farmers. For example, farmers in the pilot study had difficulties understanding the question ‘*Do you maintain any quarantine measures for newly introduced poultry into the flock?*’ and we modified the question to ‘*Do you keep newly introduced poultry separate from other poultry in a safe and separate place/house before introducing into the existing poultry flock?*’. A total of 144 backyard chicken farmers were interviewed using the farm-level questionnaire. An interview lasted about 35 min. The interviews were conducted by one female and one male trained veterinarian.

The village-level questionnaire was designed to collect information on environmental or ecological features, the village structure, types of agricultural production conducted within the village, poultry density, previous disease outbreaks and vaccination campaigns in the village. The first part of the questionnaire included 15 questions to be filled by the lead author of this paper based on observations made while walking through the village and examining environmental and agricultural village characteristics. The second part of the questionnaire included 11 questions on village demographics, poultry production and marketing within the village. The village-level questionnaire was also pilot-tested in two villages which were not included in the final study. The pilot testing resulted in the modification of five questions in the village-level questionnaire. For example, the original question, ‘*Is there a live bird market in this village?*’ was modified to ‘*Is there any market within the village where trading of poultry is conducted?*’. Answers to the second part of the village-level questionnaire were obtained through a Participatory Appraisal (PA) process. The PA was conducted as group discussions involving 5–7 key informants in each village. Key informants included the village headman, two experienced backyard chicken farmers, one Veterinary Field Assistant from the local livestock office, one school/college teacher or/and religious leader or/and a commercial poultry farmer. The key informants were contacted one week before the visit of the village. A PA lasted about 20 min.

Informed consent (signature/thumb impression) was obtained from each farmer and village key informant prior to the interview, PA and sample collection.

### Serological sampling

Chickens and ducks of Deshi breed (‘indigenous’ in Bengali) raised under free-ranging or scavenging conditions were sampled on each farm. Blood samples were collected from 4 chickens (N = 144 farms), and when ducks were present (N = 102 farms), from 2 ducks on each farm. Depending on their body weight, 1–3 ml of blood were collected from the wing or jugular vein of each bird and transferred to a sterile plastic tube immediately after collection. The tube was kept in a cool box filled with ice packs and transported to the Chattogram Veterinary and Animal Sciences University (CVASU) laboratory (for samples collected in Chattogram) and to the local office of the District Livestock Services (for samples collected in Cox’s Bazaar). Samples were refrigerated overnight; the serum was then separated by centrifugation at 10,000 rpm for 30 min at 4 °C and transferred to Eppendorf tubes.

All serum samples were further processed at the CVASU laboratory, where the samples were first screened for the presence of antibodies against Influenza A virus using commercially available Enzyme-linked Immunosorbent Assay (ELISA) kits (product code: 5004.20, IDEXX Laboratories, Inc.,USA). Influenza A positive samples were then tested for the presence of H5 and H9 specific antibodies using the Haemagglutination Inhibition (HI) test^18^. A serum sample was considered positive if there was an inhibition at a dilution of 1/16 (2^4^) or more against 4 haemagglutinating units of antigen^18^.

### Data analyses

Questionnaire data were entered in Microsoft Access 2013 databases (Microsoft Corporation, USA). Data analysis was conducted in STATA 14.1 (Stata Corporation, College Station, Texas, USA). Data analysis was conducted separately for H5 and H9.

A total of 281 farm-level and 96 village-level dichotomous and ordinal categorical variables were derived from questionnaire data. For each AIV subtype, the proportion of positive and negative farms for each risk factor was calculated.

To reduce the number of predictors, we used a correlation analysis to screen for unconditional associations^52^. Considering the dichotomous or ordinal nature of the predictors, pairwise correlations were examined by estimating the polychoric correlations coefficients^53,54^ using the -*polychoric*- command in STATA. If high correlation was identified (≥ 0.9 for H5/H9), only the most biologically plausible risk factor was kept.

Considering that farms were clustered within villages, we used mixed-effect logistic regression^55,56^ with village as a random effect to explore the association between the H5 (or H9) serological status of a flock and the potential risk factors. For categorical predictors with more than two levels, p values were computed using Wald tests (-*testparm*- command). All farm and village-level predictors associated with a p value ≤ 0.15 in the univariate analysis were further screened for pairwise correlations. Multi-variable mixed-effects logistic regression models with village as a random effect were built for each AIV subtype using a backward stepwise elimination procedure. Farm and village-level predictors were considered together in the same models. At each step, the predictors with the highest p value were removed, until all predictors remaining in the model had p values < 0.05. We also assessed for potential confounding by subsequently adding eliminated risk factors that were considered as potential confounders based on the hypothesized casual diagrams. A change in Odds Ratio (OR) > 30%^52^ for any of the predictors following the inclusion of potential confounders was considered as an indication of confounding. Biologically plausible 2-way interactions of risk factors selected in the final mixed-effect model were also explored^52^.

Furthermore, the residual Intra-class Correlation (ICC) was estimated to examine the impact of the village-level random effect, using ICC ≥ 0.05 as a ‘cutoff point’^57^. Finally, as there are not any standard measures to test the overall goodness of fit of mixed-effect model^52^, we assessed the impact of potential outliers by plotting the residuals and assessing the normality and heteroscedasticity of their distribution.

### Ethics approval and consent to participate

Ethics approval for the research was obtained from the relevant ethics committees at the University of Queensland: animal ethics approval (approval umber: SVS/465/15/RVC) and human ethcis approval (approval number: 2015001703). Informed written consent was obtained from all participants (backyard farmers and PA participants) before the interviews/PA discussions and sample collection from their birds.

## Supplementary Information

Supplementary Information.

## Data Availability

All data generated or analysed in this study is included in this manuscript as Supplementary Information file.
