# Supplementary material for: Village and farm-level risk factors for avian influenza infection on backyard chicken farms in Bangladesh
Source: Sci Rep. 2022 Jul 29;12:13009. doi: 10.1038/s41598-022-16489-5 (PMC9338044; doi:10.1038/s41598-022-16489-5)
Supplement: Supplementary file 1 — Supplementary Information. [file 41598_2022_16489_MOESM1_ESM.pdf]

Village and farm-level risk factors for avian influenza infection on backyard chicken farms in Bangladesh

Suman Das Gupta, Brishti Barua, Guillaume Fournié, Md. Ahasanul Hoque, and Joerg Henning

Risk factors (Listed in risk groups)	Category	Univariate analysis			
		H5 positive (%)	H5 negative (%)	H5 OR (95% CI)	H5 *P*-value
**Village-level factors (N=144 farms, N=42 villages)**					
Environmental or ecological features					
Migratory wild birds visiting the village	No	14 (18.4)	62 (81.6)	Reference	**0.009**
	Yes	26 (38.2)	42 (61.8)	2.7 (1.3-5.9)	
Forest or jungle present in the village	No	23 (33.8)	45 (66.2)	Reference	**0.128**
	Yes	17 (22.4)	59 (77.6)	0.6 (0.3-1.2)	
Village structure					
Pond/river/lake/canal present between households in the village	No	10 (19.6)	41 (80.4)	Reference	**0.108**
	Yes	30 (32.3)	63 (67.7)	2.0 (0.9-4.4)	
Estimated distance between the village and the closest main road	>3.5 km	5 (14.3)	30 (85.7)	Reference	**0.047**
	≤3.5 km	35 (32.1)	74 (67.9)	2.8 (1.0-7.9)	
Poultry density					
At least one commercial poultry farm present in the village	No	7 (18.4)	31 (81.6)	Reference	**0.138**
	Yes	33 (31.1)	73 (68.9)	2.0 (0.8-5.0)	
Number of households rearing backyard poultry in the village	≤300	12 (19.4)	50 (80.7)	Reference	**0.052**
	>300	28 (34.2)	54 (65.9)	2.2 (1.0-4.7)	
**Farm-level factors (N=144 farms)**					
Trading practices					
Frequency of sales of eggs, chicken or ducks within the last 12 months	0 times	8 (15.1)	45 (84.9)	Reference	**0.014**
	1 to 5 times	10 (25.6)	29 (74.4)	2.0 (0.7-5.7)	
	>5 times	22 (42.3)	30 (57.7)	4.3 (1.6-11.8)	

Risk factors (Listed in risk groups)	Category	Univariate analysis			
		H5 positive (%)	H5 negative (%)	H5 OR (95% CI)	H5 *P*-value
Number of poultry sold in the last 12 months	0	7 (18.0)	32 (82.1)	Reference	**0.064**
	1 to 30	26 (28.3)	66 (71.7)	1.9 (0.7-5.0)	
	>30	7 (53.9)	6 (46.2)	5.8 (1.3-25.2)	
Cleaning practices					
Frequency of cleaning (dry or wet cleaning) of the poultry house or places where were poultry are kept	Daily	2 (10.0)	18 (90.0)	Reference	**0.073**
	≥A Week	38 (30.7)	86 (69.4)	4.0 (0.9-18.0)	
Disposal of garbage, droppings/litter and dead birds					
Disposal of litter/droppings by throwing them into nearby rivers, lakes or canals	No	32 (25.0)	96 (75.0)	Reference	**0.046**
	Yes	8 (50.0)	8 (50.0)	3.1 (1.0-9.2)	
Visit of commercial poultry farms					
Frequency of visits of commercial poultry farms in the last 12 months by farmer or family members	0 times	29 (26.4)	81 (73.6)	Reference	**0.051**
	1 to <50 times	4 (18.2)	18 (81.8)	0.6 (0.2-2.0)	
	≥50 times	7 (58.3)	5 (41.7)	3.9 (1.2-13.3)	
Consumption of own reared poultry					
Number of home-reared poultry consumed in the last 12 months	0 to 15	39 (30.5)	89 (69.5)	Reference	**0.073**
	>15	1 (6.2)	15 (93.8)	0.1 (0.0-1.2)	

Supplementary Table S1. Results of the univariate analysis for village and farm-level risk factors (N=144 farms, N=42 villages) associated with H5 flock-level seroprevalence on backyard chicken farms in Bangladesh, 2016 that were significant at $P \leq 0.15$ (and were therefore considered for the multi-variable model) but were not significant at $P < 0.05$ in the multi-variable model.

Risk factors (Listed in risk groups)	Category	Univariate analysis			
		H9 positive (%)	H9 negative (%)	H9 OR (95% CI)	H9 *P*-value
**Village-level factors (N=144 farms, N=42 villages)**					
Village structure					
Estimated distance between the village and the closest main road	>3.5 km	13 (37.1)	22 (62.9)	Reference	**0.004**
	≤3.5 km	74 (67.9)	35 (32.1)	3.8 (1.5-9.7)	
Presence of isolated households within the village	No	16 (80.0)	4 (20.0)	Reference	**0.090**
	Yes	71 (57.3)	53 (42.7)	0.3 (0.1-1.2)	
Muddy road passing through the village	No	60 (68.2)	28 (31.8)	Reference	**0.036**
	Yes	27 (48.2)	29 (51.8)	0.4 (0.2-0.9)	
Presence of a public vehicle stop (e.g. bus, train) in the village	No	36 (52.2)	33 (47.8)	Reference	**0.090**
	Yes	51 (68.0)	24 (32.0)	2.1 (0.9-4.9)	
Poultry density					
Number of HHs rearing both chickens and ducks	<50	15 (45.5)	18 (54.6)	Reference	**0.080**
	≥50	72 (64.9)	39 (35.1)	2.5 (0.9-6.7)	
**Farm-level factors (N=144 farms)**					
Trading practices					
Farmed poultry was obtained from live bird market in the last 12 months	No	60 (54.6)	50 (45.5)	Reference	**0.015**
	Yes	27 (79.4)	7 (20.6)	3.6 (1.3-10.0)	
Number of chicken eggs sold in the last 12 months	0 to 10	62 (55.4)	50 (44.6)	Reference	**0.048**
	>10	25 (78.1)	7 (21.9)	2.7 (1.0-7.3)	
Number of visits to live bird markets to sell poultry in the last 12 months	0 times	42 (50.6)	41 (49.4)	Reference	**0.034**
	1 times	8 (80.0)	2 (20.0)	4.0 (0.7-22.1)	
	>1 times	37 (72.6)	14 (27.5)	2.6 (1.1-6.0)	
Disposal of garbage, droppings/litter and dead birds					
Disposal of dead birds by throwing them into nearby bushes/jungle	No	57 (55.9)	45 (44.1)	Reference	**0.112**
	Yes	30 (71.4)	12 (28.6)	2.0 (0.9-4.8)	

Risk factors (Listed in risk groups)	Category	Univariate analysis			
		H9 positive (%)	H9 negative (%)	H9 OR (95% CI)	H9 *P*-value
Indirect contact with other animals					
Feeding of different poultry species with the same feeder or in the same location	No	41 (53.3)	36 (46.8)	Reference	**0.033**
	Yes	46 (68.7)	21 (31.3)	2.5 (1.1-5.6)	
Pond water used as for source of drinking water for poultry	No	42 (52.5)	38 (47.5)	Reference	**0.029**
	Yes	45 (70.3)	19 (29.7)	2.7 (1.1-6.4)	
Management of outbreaks and sick birds					
Selling of sick birds at the local live bird markets	No	62 (65.3)	33 (34.7)	Reference	**0.123**
	Yes	25 (51.0)	24 (49.0)	0.5 (0.2-1.2)	
No separation of healthy chickens during disease outbreaks	No	75 (58.1)	54 (41.9)	Reference	**0.152**
	Yes	12 (80.0)	3 (20.0)	2.8 (0.7-11.6)	
Restriction of scavenging of chickens, when outbreaks occur on neigbouring farms	No	56 (65.1)	30 (34.9)	Reference	**0.090**
	Yes	31 (53.5)	27 (46.6)	0.5 (0.2-1.1)	

Supplementary Table S2. Results of the univariate analysis for village and farm-level risk factors (N=144 farms, N=42 villages) associated with H9 flock-level seroprevalence on backyard chicken farms in Bangladesh, 2016 that were significant at $P \leq 0.15$ (and were therefore considered for the multi-variable model) but were not significant at $P < 0.05$ in the multi-variable model.

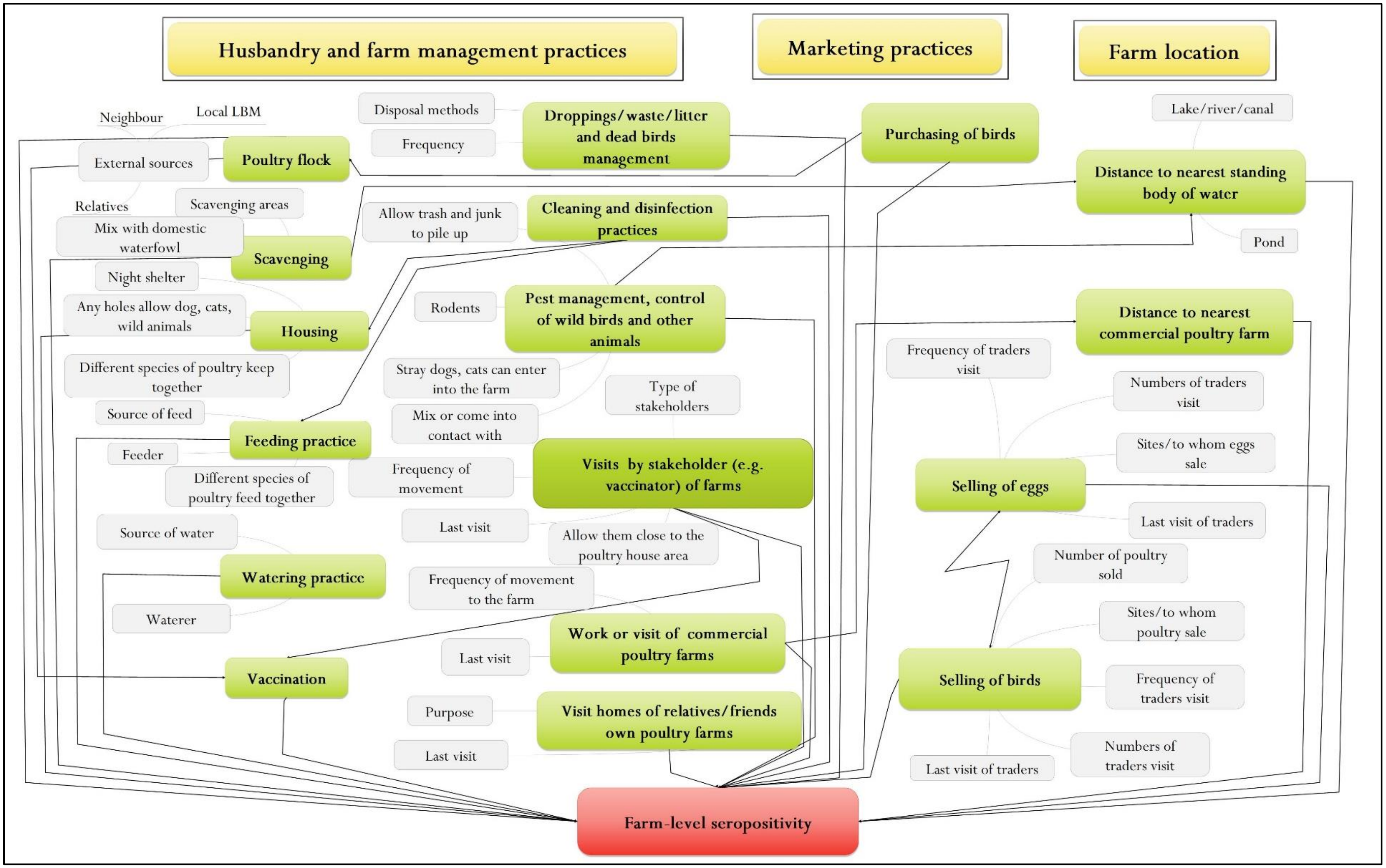

Supplementary Figure S1. Hypothesized causal pathways for farm-level risk factors (green boxes) associated with avian influenza farm-level seropositivity (red box) in backyard chickens in Bangladesh. Grey boxes indicating additional categories/levels within the risk factor. Yellow headings represent themes or categories under which risk factors can be combined. The causal pathways were used to inform the development of questions used in the interviews with backyard farmers and to guide the inclusion of potential confounders and interactions in the final multi-variable model.

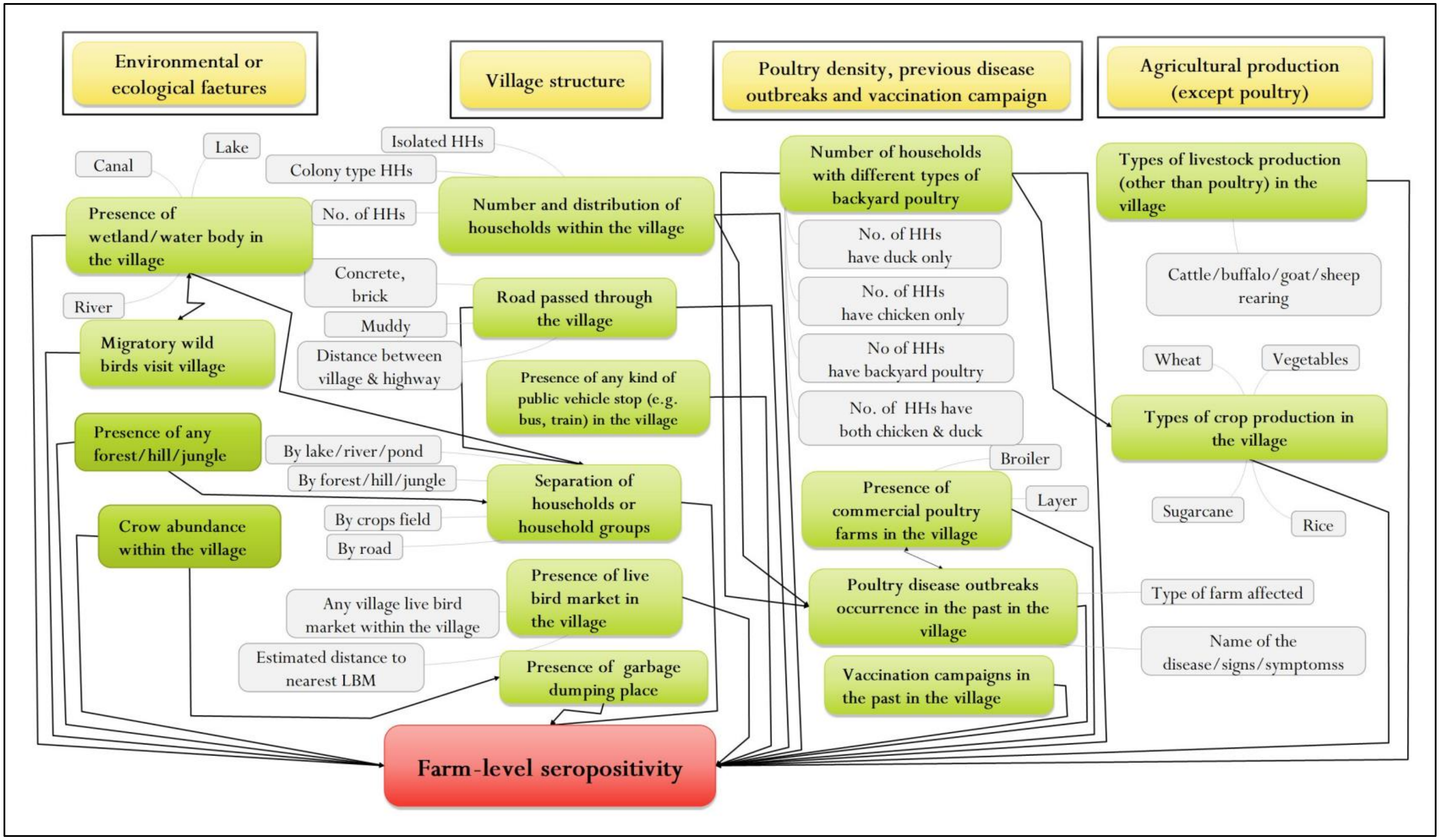

Supplementary Figure S2. Hypothesized causal pathways for village-level risk factors (green boxes) associated with avian influenza farm-level seropositivity (red box) in backyard chickens in Bangladesh. Grey boxes indicating additional categories/levels within the risk factor. Yellow headings represent themes or categories under which risk factors can be combined. The causal pathways were used to inform the development of questions used in the interviews with backyard farmers and to guide the inclusion of potential confounders and interactions in the final multi-variable model.
